# CHRONIC USE OF PROTON PUMP INHIBITORS AND THE QUANTITY OF G, D, AND ECL CELLS IN THE STOMACH

**DOI:** 10.1590/0102-672020190001e1506

**Published:** 2020-08-24

**Authors:** Silvia Maria Perrone CAMILO, Élia Cláudia de Souza ALMEIDA, Jacqueline Batista SOUSA, Luana Perrone CAMILO, Renata Margarida ETCHEBEHERE

**Affiliations:** 1 Post-Graduate Program in Health Sciences, Gastroenterology Service, Triângulo Mineiro Federal University, Clinical Hospital, Uberaba, MG, Brazil; 2 Post-Graduate Program in Health Sciences, Triângulo Mineiro Federal University, Uberaba, MG, Brazil; 3 Faculdade Israelita de Ciências da Saúde Albert Einstein, Medicine Graduation, São Paulo, SP, Brazil; 4 Post-Graduate Program in Health Sciences, Surgical Pathology Subunity, Clinical Hospital, Triângulo Mineiro Federal University, Uberaba, MG, Brazil

**Keywords:** Omeprazole, Gastrin, Somatostatin, Enterochromaffin-like cells, Helicobacter pylori, Omeprazol, Gastrina, Somatostatina, Células enterocromafim, Helicobacter pylori

## Abstract

**Background:**

Acid inhibition from chronic proton pump inhibitor use and a possible increase in gastrin can lead to changes in the regulation of hydrochloric acid production. However, it has not known whether such chronic use changes the presence of gastrin, delta, and enterochromaffin-like cells in the stomach or the relationship between gastrin and delta cells.

**Aim:**

To analyze the number of gastrin-producing gastrin cells, somatostatin-producing cells, and histamine-producing cells in patients who were chronic users of proton pump inhibitor, with or without related *Helicobacter pylori* infection.

**Methods:**

Biopsies from 105 patients, including 81 chronic proton pump inhibitor users (experimental group) and 24 controls, were processed immunohistochemically and subjected to counting of gastrin, delta, and enterochromaffin-like cells in high-magnification microscopic fields and in 10 glands.

**Results:**

Gastrin cell, delta cell, and enterochromaffin-like cells counts were similar across the groups and appeared to be unaffected by *Helicobacter pylori* infection. The ratio between gastrin cells and delta cells was higher in the chronic users of proton pump inhibitor group than in controls.

**Conclusion:**

Chronic users of proton pump inhibitor does not affect gastrin cell, delta cell, and enterochromaffin-like cell counts significantly, but may alter the ratio between gastrin cells and delta cells.

## Graphical Abstract

[Fig f1a]

**Figure f1a:**
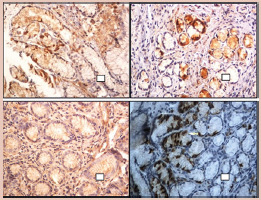
IHC identifying G, D, and ECL cells

## RESUMO

**Racional:**

A inibição ácida pelo uso crônico de inibidores de bomba de prótons e o possível aumento da gastrina podem ser seguidos de alterações na regulação da produção do ácido clorídrico. Ainda não está definido se o uso crônico altera a quantidade de células G, D e ECL no estômago ou a razão células G/D.

**Objetivo:**

Avaliar o número de células G - produtoras de gastrina -, células D - produtoras de somatostatina - e células ECL - produtoras de histamina -, em pacientes com uso crônico de inibidores de bomba de prótons, com ou sem infecção pelo *Helicobacter pylori*.

**Método:**

Trata-se de estudo retrospectivo avaliando 105 pacientes, 81 usadores crônicos de inibidores de bomba de prótons e 24 controles, através de biópsias com contagem das células G, D e ECL por estudo imunoistoquímico, de forma quantitativa onde havia maior número de células positivas por campo microscópico de grande aumento e em 10 glândulas.

**Resultados:**

Não houve diferença estatística comparando-se o número de células G, D e ECL. A razão entre as células G e D foi maior nos pacientes usadores crônicos de inibidores de bomba de prótons.

**Conclusão:**

O uso crônico de inibidores de prótons parece não interferir na contagem das células G, D e ECL, porém, interfere na razão entre as células G e D.

## Graphical Abstract

[Fig f1as]

**Figure f1as:**
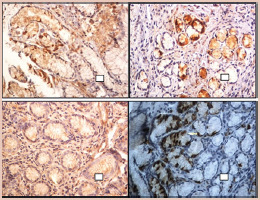
Positividade na imunoistoquímica para as células G, D e ECL

## INTRODUCTION

Acid secretion plays an important role in regulating gastric functions. It protects against pathogenic gastrointestinal agents, facilitates digestion and the absorption of some nutrients, and modulates eating behavior^17^ ^,^ ^22^.

Hydrochloric acid (HCl) is produced in two main areas in the stomach: the oxyntic mucosa and the antral mucosa. Parietal cells, which make up some 80% of the acid-secreting gastric mucosa, are located in the oxyntic mucosa. They are found proximally in the gastric body and fundus, intermingled with histamine-secreting enterochromaffin-like cells (ECL) and the main pepsinogen-secreting cells. The distal mucosa, which makes up about 20% of the gastric mucosa, contains antral pyloric glands with G cells, which secrete gastrin. Both areas include surface mucosa cells, colon mucosa cells, and enterochromaffin cells, as well as D cells, which secrete somatostatin^11^ ^,^ ^14^ ^,^ ^19^ ^,^ ^24^.

Regulation of gastric acid secretion is achieved through an interaction between the two main gastric endocrine cells, the G cells and the D cells. When food enters the stomach, the protein component stimulates G cells to release gastrin, which stimulates ECL cells and parietal cells to release histamine and HCl, respectively. As the acidity of the stomach and duodenum increases, protective feedback mechanisms are activated to inhibit further secretion of HCl. An important inhibitory control mediated by HCl involves the release of somatostatin by D cells^17^.

The acid inhibition that occurs with the chronic use of proton pump inhibitors (PPIs) and possible associated increases in gastrin levels may lead to changes in the mechanisms regulating HCl production. However, it is unclear whether their chronic PPI use changes the quantities of G, D, and ECL cells, or the ratio between G cells and D cells, in these patients. It is also unclear whether *Helicobacter pylori* (HP) infection has an influence on these changes.

So, the aim of this research was to analyze the number of gastrin-producing cells, somatostatin-producing cells, and histamine-producing cells in patients who were chronic users of proton pump inhibitor, with or without related *Helicobacter pylori* infection 

## METHODS

We performed a retrospective study at a public tertiary hospital that was approved by the institution’s Research Ethics Committee (CAAE: 63812217.1.0000.5154). The participants signed informed consent forms.

Initially, a total of 105 patients were evaluated, including 81 chronic PPI users (PPI group) and 24 non-users (control group). Chronic PPI use was defined as six months of continuous use. However, in some cases, it was not possible to perform the immunohistochemical evaluation of all three antibodies. The inclusion criteria were: willingness to participate in the study and completion of an intake questionnaire. The exclusion criteria were any prior surgery on the digestive tract or pernicious anemia.

All patient participants underwent a medically indicated upper digestive tract endoscopy conducted with an Olympus^®^ (GIF-Q150 and GIF-2T160) video gastroscope linked to an Exera^®^-CLV-160 processor. Before conducting each endoscopic examination, 4 ml of blood was collected for gastrin level determination. Gastrin was measured through chemiluminescence, with the results expressed in pg/ml (normal reference range, 13-115 pg/ml). Patients gastrin levels greater than 500 pg/ml were referred for investigation into the causes of their hypergastrinemia^25^. Samples of mucosa from the antral and gastric body areas were collected as medically indicated during the endoscopic procedure.

The samples were fixed immediately in 4% buffered formaldehyde, embedded in paraffin, and sectioned into 3 µm-thick sections on a microtome. The sections were mounted on slides, which were subjected to H&E staining for general histology, Warthin-Starry staining for identifying bacteria with an *H. pylori*-consistent morphology, or immunohistochemistry (IHC) to quantitate G cells, D cells, and ECL cells. For the IHC, we used a polymer-based detection technique with antibodies targeting G cells (LSBIO^®^-USA), D cells (SANTA CRUZ^®^-USA), and histidine decarboxylase (HDC; PROGEN^®^-USA), a specific histamine-producing enzyme^23^ ^,^ ^29^.

Immunolabeled cells were located and quantified in the antrum (anti-G cell and anti-D cell immunopositivity) and in the gastric body (anti-D cell and anti-HDC immunopositivity) with a Nikon^®^ ECLIPSE 80*i* regular optical microscope. We counted all immunopositive cells within a 400× high magnification field, where immunopositivity for each antibody was abundant, as well as in a sample of 10 glands showing large numbers of immunopositive cells. We analyzed G cell, D cell, and ECL cell (i.e. HDC-immunopositive) counts, serum levels of gastrin, and the presence vs. absence of HP infection.

### Statistical analyses

Were performed in BIOSTAT^®^ on a database developed in Excel^®^ 2010. The tests were considered significant when the probability of rejecting the null hypothesis was below 5% (p< 0.05).

## RESULTS

The general characteristics of the sample are shown in Table 1. There were no significant demographic differences between the PPI and control groups. A majority of the 81 patients (53, 65.4%) used omeprazole at a daily dose of 20 mg, whereas 23 (28.4%) took 40 mg/day of omeprazole and three (3.7%) took 60 mg/day of omeprazole. The remaining two patients used 40 mg/day of pantoprazole. With respect to length of use, most of the 81 PPI group patients (72, 88.9%) had been using a PPI for >12 months, the remaining patients (9, 11.1%) had been using a PPI for 6-12 months.

**TABLE 1:** Age, gender, race, and serum level of gastrin in patients who were chronic PPI users and non-user controls

Variable	Control (n=24)	PPI (n=81)
Age, years	48.5 (17-72)	57 (23-79)
Gender, women/men	70.8% / 29.2%	84% / 16%
White	66.6%	76.5%
Non-white	33.3%	23.5%
Increased gastrin*	16.7%	34.6%

*Serum gastrin= >115 pg/ml; all p >.05 control vs. PPL.

We examined gastric antrum samples from 15 control group participants, the majority of whom (9, 60%) were HP negative. We examined gastric body samples from 21 control group participants, the majority of whom (14, 66.6%) were also HP negative. We examined gastric antrum samples from 54 PPI group patients, including 34 HP negative (63%) and 20 HP positive (37%) patients. We examined gastric body samples from 70 PPI group patients, including 42 HP-negative (60%) and 28 HP-positive (40%). The G cell, D cell, and ECL cell counts obtained in the antrum and the gastric body for the PPI and control groups are reported in Table 2.

Participants with HP infection and participants without it had similar G cell, D cell, and ECL cell counts as well as similar serum gastrin levels. We therefore analyzed all PPI group participants and all control participants together as unified groups (Table 3).

**TABLE 2:** G cell, D cell, and ECL cell counts* in the gastric antrum and gastric body of chronic PPI users and non-user controls, with and without HP infection

Cell type	PPI group		Control group	
	HP positive	HP negative	HP positive	HP negative
Gastric antrum	n=20	n=34	n=6	n=9
G 10 glands	47.5 (14-74)	46 (24-108)	35.5 (28-43)	42 (23-75)
G field	73 (14-166)	80 (20-296)	57 (34-72)	59 (20-100)
D 10 glands	22.5 (15-32)	24.5 (12-35)	40 (33-56)	42 (21-57)
D field	28.5 (14-59)	34 (13-79)	59 (43-81)	57 (45-112)
Gastric body	n=28	n=42	n=7	n=14
ECL field	5 (1-11)	4 (1-12)	4 (1-12)	5 (1-14)
D 10 glands	16.5 (9-30)	17 (10-28)	15 (13-25)	17.5 (13-26)
D field	16.5 (9-55)	19.5 (9-47)	19 (8-32)	19.5 (10-36)

*Fisher’s exact tests showed all of the above PPI vs. control comparisons were not significant, p >.05

**TABLE 3:** G cell, D cell, and ECL cell counts in the gastric antrum and gastric body by group, as determined by two sampling methods

Cell type	Antrum					Body			
	Control n=15		PPI n=54			Control n=22		PPI n=69	
	10 glands	Field	10 glands	Field		10 glands	Field	10 glands	Field
G	38	59	94	79		-	-	-	-
D	42	57	24	31		18	20	17	18.5
ECL	-	-	-	-		-	5	-	4

*G cell, D cell, and ECL cell counts were similar in PPI and control groups in both areas (Fisher’s exact tests all p>.05)

**FIGURE 1:**
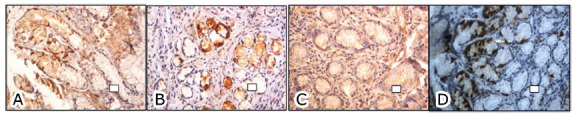
A) Gastric antral mucosa from a control patient, anti-G cell immunopositivity in brown; B) Gastric antral mucosa from a control patient, anti-D cell immunopositivity in brown; C) Gastric oxyntic mucosa, anti-ECL cell immunopositivity in brown; D) Gastric antral mucosa of a control patient, anti-G cell immunopositivity in brown (arrow).

IHC identifying G, D, and ECL cells are shown in Figure 1. We observed a G cell to D cell ratio in the antrum of 3.9 (94:24) for the PPI group based on counts in 10 sampled glands and a G cell to D cell ratio of 2.5 (79:31) for the PPI group based on counts in a high-magnification field. In the control group, the G cell to D cell ratios found were 0.9 (38/42) for the 10 sampled glands and of 1 (59/57) for the high-magnification field.

## DISCUSSION

PPIs are a very widely used class of medications, but they are often used for indications and treatment durations that are not well tested or approved, and they can be acquired without prescription in some countries^31^. It is estimated that up to 10% of the population worldwide uses some type of PPI ^30^. They are the strongest suppressors of gastric acid secretion that are independent of other stimulatory factors. At commonly used dosages, PPIs reduce daily HCl production (basal and stimulated) by 80-95%^11^.

PPI therapy can lead to an increase in serum levels of gastrin^2^ ^,^ ^7^ ^,^ ^16^. Hypergastrinemia occurs through a negative feedback mechanism between the intragastric pH and antral G cell activity^16^. In some patients, hypergastrinemia is thought to be responsible for the HCL rebound hypersecretion that occurs after PPI withdrawal^30^. However, gastrin levels generally return to normal after interruption of PPI use^26^.

According to Arnold and Koop^2^, although serum gastrin increases over the short- and long-term with PPI treatment, it is rare that gastrin levels exceed those observed after a proximal selective vagotomy and occurs when the inhibition of the stimulus to acid secretion exceeds 80%. These authors point out that in studies of patients receiving omeprazole at doses of 40-60 mg/day for a period of 1-2 years, moderate gastrin increases are observed with inter-individual variation. Cheung and Leung^6^ indicated that gastrin levels are increased in patients who have used PPIs for more than three years. The lack of a significant inter-group gastrin difference in our study may be explained by the use of a relatively lower PPI dose (i.e. 20 mg/day) by the majority of participants and by a period of generally <3 years.

Hypergastrinemia could not be attributed to gastrinoma, autoimmune gastritis, pyloric obstruction, renal insufficiency, surgery (vagotomy or retained gastric antrum following Billroth II gastrectomy)^4, 21^ **,** or chronic atrophic gastritis^5^ in any of our patients. However, four patients with altered gastrin in our PPI group were seropositive for Chagas disease, which has been shown to cause hypergastrinemia^20^ ^,^ ^28^.

A study performed in rats showed that omeprazole use led to a significant increase in G-cell density combined with a reduction in D-cell density and, consequently, a marked increase in the G/D cell ratio^16^. These observations suggested that the G/D cell ratio in the antrum may be governed by intragastric pH. However, changes in this magnitude have not been observed in humans^16^. As with our study, Arnold and Koop^2^ did not find differences in G cell or D cell counts, nor in ECL cell density, in patients with severe reflux esophagitis treated with 40 mg/day of omeprazole for up to nine months; they did not indicate the HP infection status of their patients. Patients infected with strains of HP that express CagA, a cytotoxin encoded by the A gene that acts as a virulence factor strongly associated with severe gastritis have been found to have a reduced D-cell density in the antrum with no changes in the number of G cells^15^.

We observed a higher G/D cell ratio in the antrum of patients in the PPI group than in the control group, despite a lack of difference in cell counts when we compared them separately. An increase in the G/D cell ratio indicates a functional disturbance in the gastrointestinal tract and can be observed in patients with gastritis, ulcerated mucosal lesions, or HP infection^3^. D cell density is significantly lower in patients infected by HP than in those who are not infected and in normal individuals, while the G cell density is significantly increased. An association with the severity of inflammation caused by the release of cytokines might explain these changes by way of an influence on gastrin/somatostatin homeostasis and might be related to the virulence of infecting HP strain^17^.

Liu *et al* ^17^ found a G/D cell ratio of 3 in people who did not use a PPI. Likewise, Frick *et al* ^10^ found a G/D cell ratio of 3 in normal rat stomachs. These values are similar to those found in our study. On the other hand, Lamberts *et al* ^16^ described a G/D ratio of 5.5 in the antral mucosa before and after treatment with omeprazole at doses in the range of 40-60 mg/day for 1-2 years. However, their patients were using relatively high PPI doses and they employed a cell counting method different from that used here.

Because gastrin stimulation of histamine release from ECL cells is the main trigger of gastric acid secretion, with ECL cells being the main receiver of the gastrin signal rather than the HCl-secreting parietal cells themselves^30^, we were interested to determine if ECL cell counts may be increased in patients who were chronic PPI users. Gastrin is characterized by its trophic capacity ^8^ ^,^ ^18^. Reports of ECL cell hyperplasia and gastric carcinoids in 45% of rats treated for two years with 140 pg/kg omeprazole raised doubts about the safety of omeprazole in humans. However, the dosage used in the animals was approximately 500 times the dose currently used in humans, and the rats were treated throughout their lives^1^ ^,^ ^16^.

Changes in gastric cell counts and a possible relationship between neuroendocrine tumors and chronic PPI use have been examined with respect to the period of use required for these changes to occur and what factors might aggravate or accelerate this process^16^. The higher incidence of carcinoid tumors in patients with pernicious anemia^16^ suggests that achlorhydria is followed by high levels of gastrin in these patients. It has been a challenge to define whether patients treated for a long period of time with omeprazole can be compared to those with pernicious anemia^16^.

Liu *et al* ^17^ reported that ECL cells in the gastric body mucosa constitute an important proportion of endocrine cells. However, the exact percentage of these cells have yet to be defined. We found small ECL cell populations in the oxyntic mucosa in both groups, without a statistically significant difference between their quantities. Lamberts *et al* ^16^ described two studies with differing results in terms of increases in the number of argyrophil cells after one year of omeprazole treatment. Creutzfeldt *et al* ^7^ found that the number of argyrophil cells in the oxyntic mucosa had increased in a sample of 10 patients after taking omeprazole for up to two years, relative to their pre-treatment levels. This increase occurred soon after one year and was attributed to argyrophil cell hyperplasia. No further increases were seen after two years. However, no such increase was found in a larger group of 18 patients studied before and after one year of omeprazole treatment^7^. The authors attributed this difference to different pretreatment values and possible errors caused by the small sample of gastric biopsies examined^7^. Traditional histochemical techniques (argyrophilia) have much lower specificity than IHC^23^ ^,^ ^29^. IHC techniques with specific antibodies against products secreted by endocrine cells have thus replaced older histochemical techniques^27^.

Liu *et al* ^17^ have noted a lack of studies reporting exact percentages of ECL cells in the gastric body. Using antibodies for gastrin, somatostatin, and chromogranin, they found higher proportions of ECL cells in the gastric body than elsewhere in the stomach. Arnold and Koop^2^ noted that ECL cells are almost exclusively restricted to the fundic mucosa and account for 40-60% of the endocrine cells in this part of the stomach. The number of ECL cells found in the gastric body in our study using anti-HDC IHC was lower than the numbers of D cells found in the control group and in the PPI group, with or without HP infection. The HDC enzyme converts histidine into histamine^9^ ^,^ ^12^ ^,^ ^13^. HDC is also expressed in mastocytes and basophils, wherein histamine is stored in granules^12^, these are cells with different morphological characteristics than ECL cells.

Another marker of endocrine cells is chromogranin A. Chromogranin A, an acid protein located in ECL cells, is a biosynthetic precursor of several bioactive peptides^9^. Chromogranin A expression is used for ECL cell quantitation by subtracting somatostatin- and serotonin-producing cells from the total number of labelled cells to obtain an ECL cell count^14^. Kakehasi^14^ reported that ECL cells appear to constitute 30-40% of the cells in the oxyntic mucosa when identified using chromogranin A. Thus, the lower number of ECL cells found in our study likely stems from our use of a marker that was more specific to these cells, namely HDC.

Our ability to compare our findings with published results is hindered by the different methods of cell counting that have been used and the associated lack of methodological standardization. Variance among reports may also stem from differences in sample collection. According to Öberg^27^ and Liu *et al* ^17^, because ECL cells are located mainly in the middle- and lower-third of the mucosal bed, they may not be visible in superficial biopsies. To minimize this bias, we counted cells in 10 glands in the deepest portion of the mucosa.

Longer-term studies, especially including patients who have used proton pump inhibitor for >3 years, are needed to define the period of use that may lead to significant shift in stomach mucosal cell populations.

## CONCLUSION

We did not observe alterations in G cell, D cell, or ECL cell counts in patients who were chronic PPI users, relative to non-user controls, after 12 months of use. However, these patients did show altered G/D cell ratios. Notwithstanding, studies with patients who have been using PPIs for more than three years are needed to determine whether longer periods of PPI use might disrupt the numbers of these cells or the ratios between them. It also remains to be determined whether these cell amounts are sensitive to infection with highly virulent strains of HP or inflammation of severe intensity.
